# *Syngonanthus nitens* Bong. (Rhul.)-Loaded Nanostructured System for Vulvovaginal Candidiasis Treatment

**DOI:** 10.3390/ijms17081368

**Published:** 2016-08-22

**Authors:** Matheus Aparecido dos Santos Ramos, Luciani Gaspar de Toledo, Giovana Maria Fioramonti Calixto, Bruna Vidal Bonifácio, Marcelo Gonzaga de Freitas Araújo, Lourdes Campaner dos Santos, Margarete Teresa Gottardo de Almeida, Marlus Chorilli, Taís Maria Bauab

**Affiliations:** 1 Department of Biological Sciences, School of Pharmaceutical Sciences, UNESP-Univ Estadual Paulista, Araraquara, São Paulo 14800-903, Brazil; matheusramos_91@hotmail.com (M.A.d.S.R.); lucianitoledo@hotmail.com (L.G.d.T.); brunavidalb@gmail.com (B.V.B.); 2 Department of Drugs and Medicines, School of Pharmaceutical Sciences, UNESP-Univ Estadual Paulista, Araraquara, São Paulo 14800-903, Brazil; giovana.calixto@gmail.com (G.M.F.C.); chorilli@fcfar.unesp.br (M.C.); 3 Federal University of São João Del-Rey, Divinópolis, Minas Gerais 36307-352, Brazil; mgfaraujo@ufsj.edu.br; 4 Department of Organic Chemistry, Chemisty Institute, UNESP-Univ Estadual Paulista, Araraquara, São Paulo 14800-060, Brazil; loursant@gmail.com; 5 Department of Infectious Diseases, Faculty of Medicine of São José do Rio Preto-FAMERP, São José do Rio Preto, São Paulo 15090-900, Brazil; margarete@famerp.br

**Keywords:** liquid crystal precursor system, *Candida albicans*, *Syngonanthus nitens*, vulvovaginal candidiasis, therapeutic treatment

## Abstract

Herbal-loaded drug delivery nanotechnological systems have been extensively studied recently. The antimicrobial activity of medicinal plants has shown better pharmacological action when such plants are loaded into a drug delivery system than when they are not loaded. *Syngonanthus nitens* Bong. (Rhul.) belongs to the Eriocaulaceae family and presents antiulcerogenic, antioxidant, antibacterial, and antifungal activity. The aim of this study was to evaluate the antifungal activity of *Syngonanthus nitens* (*S. nitens*) extract that was not loaded (E) or loaded (SE) into a liquid crystal precursor system (S) for the treatment of vulvovaginal candidiasis (VVC) with *Candida albicans*. The minimal inhibitory concentration (MIC) was determined by the microdilution technique. Additionally, we performed hyphae inhibition and biofilm tests. Finally, experimental candidiasis was evaluated in in vivo models with *Wistar* female rats. The results showed effective antifungal activity after incorporation into S for all strains tested, with MICs ranging from 31.2 to 62.5 μg/mL. Microscopic observation of SE revealed an absence of filamentous cells 24 h of exposure to a concentration of 31.2 μg/mL. E demonstrated no effective action against biofilms, though SE showed inhibition against biofilms of all strains. In the in vivo experiment, SE was effective in the treatment of infection after only two days of treatment and was more effective than E and amphotericin B. The *S. nitens* is active against *Candida albicans* (*C. albicans*) and the antifungal potential is being enhanced after incorporation into liquid crystal precursor systems (LCPS). These findings represent a promising application of SE in the treatment of VVC.

## 1. Introduction

Microorganisms of the genus *Candida* are associated with shallow, deep and systemic infections, particularly in patients with underlying diseases such as diabetes and in immunosuppressed patients, patients with neutropenia and transplant patients, due to the opportunistic profile presented by the members of this genus [1]. The *Candida albicans* (*C. albicans*) species is presented as the main agent of opportunistic fungal diseases that affect women of all ages, such as vulvovaginal candidiasis (VVC). The pathogenicity of *C. albicans* species involves mechanisms of aggression attributed to virulence factors. These mechanisms include the ability to defend itself against the host immune system, hyphae proliferation, biofilm formation in tissue or on medical devices, and production of harmful hydrolytic enzymes including proteases, phospholipases and hemolysin [2,3,4].

Drug therapy targeting fungal infections has limitations such as the high cost of drugs available in the clinic, high toxicity, drug interactions, insufficient bioavailability of the active ingredient and the emergence of resistant strains [5]. Several antifungals have been indicated for the treatment of these infections, including those belonging to the polyenic, azole, and echinocandin classes; however, due to the indiscriminate use of these antimicrobials targeting genetic and physiological characteristics of the fungus, there has been a significant increase in profile resistance to drug members of these classes [6,7]. The health sciences have been increasingly concerned with the exaggerated growth of multidrug-resistant strains to antibiotics currently in use in clinical practice. Therefore, medicinal plants have emerged in the search for new antifungal drugs due to their demonstration of important bioactive properties and the presence of secondary metabolites such as tannins, flavonoids and phenols [8].

Several plants have been studied to investigate their antimicrobial potential. Some families of plant species have been featured in science, such as Eriocaulaceae. Eriocaulaceae are popularly known as “evergreens” and are used in the manufacture of decorative ornaments and accessories [9]. Among these is the genus *Syngonanthus*, which is found in the states of Goiás and Tocantins, Brazil, where it is known as “golden grass” [10,11]. The species *Syngonanthus nitens* (Bong.) Ruhland has received substantial attention recently. Pacifico et al. [12] analyzed the scapes characteristics and found approximately 17 compounds derived from flavones and apigenin plus six new novel molecules. Araújo et al. [13] detected a significant potential antifungal present in the scapes of *Syngonanthus nitens* (*S. nitens*) that exhibited activity against four species of the genus *Candida*, which is a characteristic therapeutic profile of plant species when subjected to an experimental model of VVC. Although the use of natural products in screening for new drugs is very important, certain limitations have been observed in the development of clinical and pharmacological investigations. These include the difficulty of solubilizing the sample to be analyzed, which may reduce its bioavailability. In this context, nanotechnological tools are commonly used to establish secure platforms for the placement of drugs to develop drug delivery systems. Microemulsions, polymer nanoparticles, solid lipid nanoparticles, liposomes, and, more recently, liquid crystals have been used to enhance therapeutic action and drug selectivity in the vaginal environment [14].

The liquid crystal precursor systems (LCPS) are classified as promising alternatives for the treatment of vulvovaginal candidiasis. Their features are interesting for vaginal administration because they may include liquid forms that facilitate the administration of the formulation. However, upon contact with the vaginal mucosa, LCPS has the ability to incorporate water from the vaginal mucus, thereby becoming a liquid-crystalline mesophase that can promote more viscous controlled release and result in greater applicability of the components of the vegetal extract [15,16].

Recently, the biological activity of *S. nitens* was evaluated by our research group in an investigation of prophylactic potential against vaginal infection caused by *Candida krusei* [17]. After incorporation in a similar system, it was found that the system is able to enhance the action of the plant extract in all in vitro biological assays as well as promoting prevention of infection by this fungal species according to the model of in vivo infections. In this sense, we propose in this study to investigate the antifungal potential of the methanolic extract of scapes of *S. nitens* loaded (or not) into a new liquid crystal precursor system, to highlight their biological applicability for the treatment of vulvovaginal candidiasis caused by *C. albicans* azole-resistant derivatives.

## 2. Results

### 2.1. Development of Liquid Crystal Precursor Systems (LCPS)

#### 2.1.1. Preparation of the Ternary Phase Diagram

**Figure 1:**
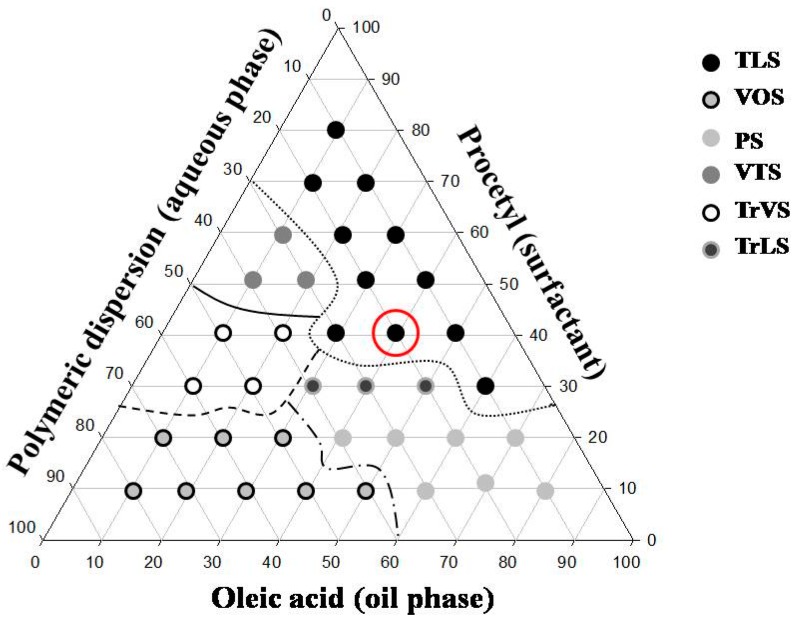
Ternary phase diagram with procetyl, oleic acid, and polymeric dispersion. The dashed lines represent the different regions of the diagram. The formulation S (chosen for to study) is highlighted in red. TLS = Transparent Liquid System; TrLS = Translucent Liquid System; TrVS = Translucent Viscous System; VOS = Viscous and Opaque System; PS = Phase Separation; VTS = Viscous Transparent System.

Figure 1 shows the 36 formulations obtained from the mixture of the system components.

The purpose of choice of the components that comprise the system was due to the possibility of obtaining an adhesive formulation and a possible interference in the membrane of fungal cell.

A transparent liquid system (S) composed of 40% *w*/*w* oleic acid (OA), 40% Procetyl (PRO) and 20% *w*/*w* polymer dispersion (PD) was selected as the liquid crystal precursor system because its liquid phase facilitated its vaginal administration by syringe.

#### 2.1.2. Assay with Artificial Vaginal Mucus (AVM)

Table 1 shows the results of analysis of the Polarized Light Microscopy (PLM) mesophases found for each proportion of Artificial Vaginal Mucus (AVM) added to formulations.

The viscosity of formulation S (formulation without extract) increased in a manner that was directly proportional to the concentration of AVM, demonstrating that the greater the amount of AVM, the higher the system viscosity. The same procedure was repeated with SE (formulation with extract) and the same results were found; thus, the incorporation of the extract into the system did not interfere structurally with the precursor behavior of the liquid crystals.

**Figure 2:**
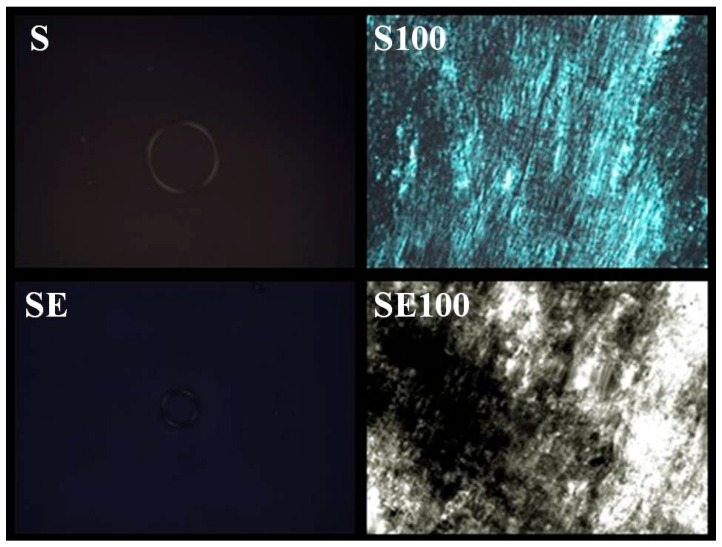
Analyses by PLM of the formulations before and after addition of AVM (20× magnification). Legend: S = Formulation without extract; SE = Formulation with extract; S100 = Formulation + 100% of AVM; SE100 = Formulation with extract + 100% of AVM.

The results obtained by PLM (Figure 2) showed that the transition of S to S100 modified the structure. S no longer possessed the characteristic of microemulsion and began to display hexagonal structures. This transition proved that S had the profile of a liquid crystal precursor. The same data were obtained with SE. In this sense, these results showed that there exists no difference after the incorporation of the extract.

#### 2.1.3. Rheological Analysis

**Figure 3:**
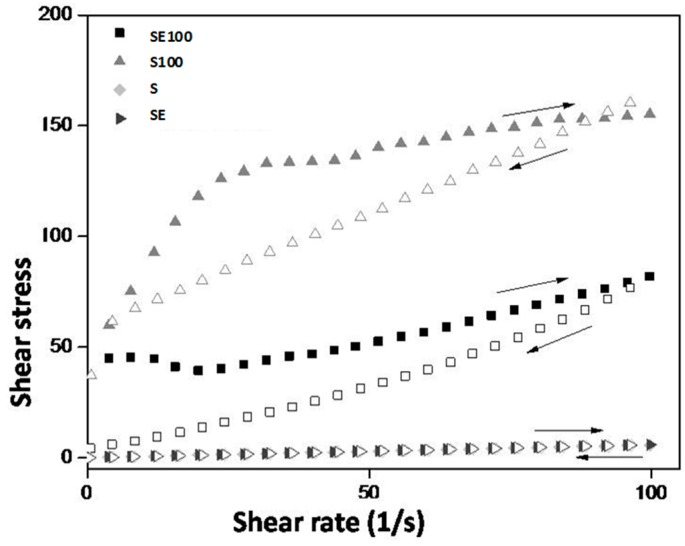
Rheogram of the formulations. Filled symbol upslope and downslope open symbol. Legend: S = Formulation; SE = Formulation with extract; S100 = Formulation + 100% of AVM; SE100 = Formulation with extract + 100% of AVM; Up Arrow = ascending; Down Arrow = descending.

The continuous rheological behavior data were plotted on a shear rate (Pa) versus shear stress (1/s) graph, as shown in Figure 3. The results demonstrate ascending and descending curves that indicate the flow behavior of the formulations.

Both formulations without AVM (S and SE) behave as a Newtonian liquid (*n* = 1) without thixotropy in which shear stress is directly proportional to the shear rate. Additionally, the descending curve overlaps with the ascending curve because their initial structure has not been changed with shear, which is characteristic of microemulsion [18].

These formulations also showed a low viscosity, as indicated by the low consistency index (K) in Table 2. Therefore, the incorporation of E did not change the flow behavior of the liquid crystal precursor and both S and SE had a flow property that rendered them easily applicable to the required site with a syringe.

However, when these formulations (S and SE) came into contact with the AVM, the resulting diluted formulations (S100 and SE100) underwent a change in flow behavior (also shown in the graph of Figure 2). These formulations had a pseudoplastic behavior (*n* < 1) with thixotropy (i.e., they were able to return to their original structures when the shear stress was removed) and showed an increase in their consistency indices (K). This type of behavior is characteristic of liquid crystalline systems due to the formation of the semisolid crystalline structure [19].

#### 2.1.4. In Vitro Evaluation of Mucoadhesive Force

**Figure 4:**
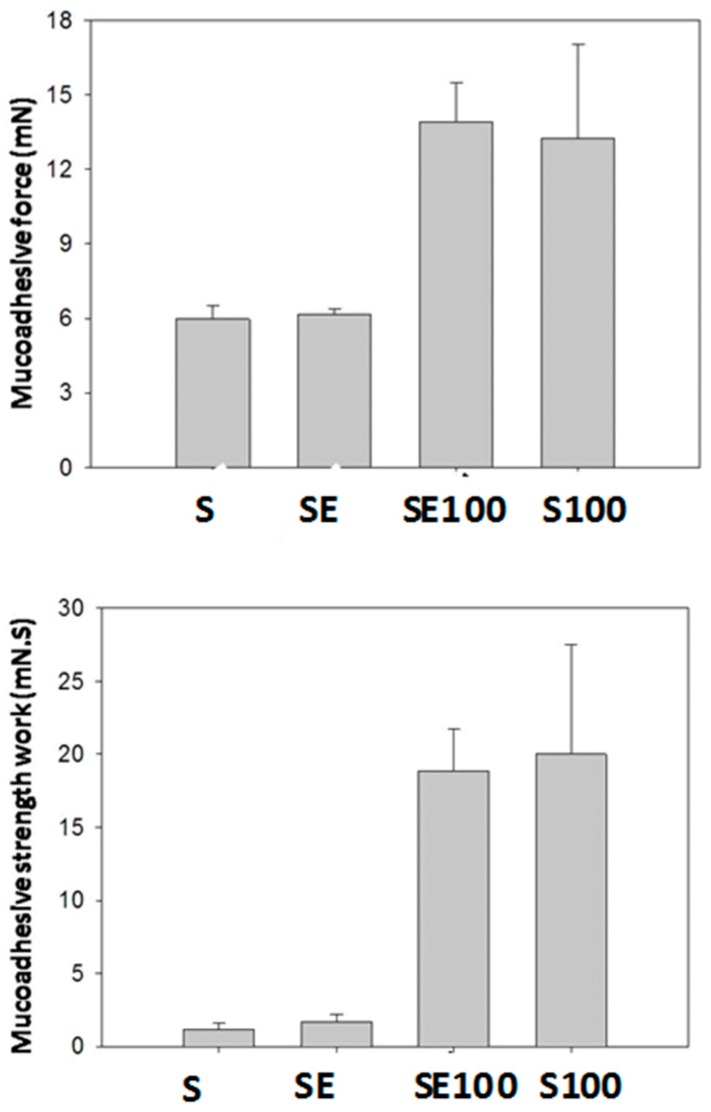
Parameters of in vitro bioadhesion test of all formulations. Each value represents the mean (±SD) of at least seven replicates. Legend: S = Formulation; SE = Formulation with extract; S100 = Formulation + 100% of AVM; SE100 = Formulation with extract + 100% of AVM.

Figure 4 illustrates the mucoadhesive strength and the mucoadhesive strength of all formulations. The data were collected at 37 ± 0.5 °C.

The results showed that the incorporation of the extract did not cause significant changes in the mucoadhesion of the formulations (*p* > 0.05). However, when the liquid crystal precursor compositions (S and SE) were diluted by the artificial vaginal mucus, there was a significant increase in the mucoadhesive parameters. 

### 2.2. In Vitro Antifungal Assays

#### 2.2.1. Determination of the Minimum Inhibitory Concentration (MIC)

Table 3 showed the minimal inhibitory concentration (MIC) results obtained in the evaluation of the antifungal activity of E and SE against all strains used in the study and the results obtained with the antifungal agents used as positive controls.

#### 2.2.2. Analysis of the Inhibitory Profile of Hyphae in *Candida albicans* (*C. albicans*)

**Figure 5:**
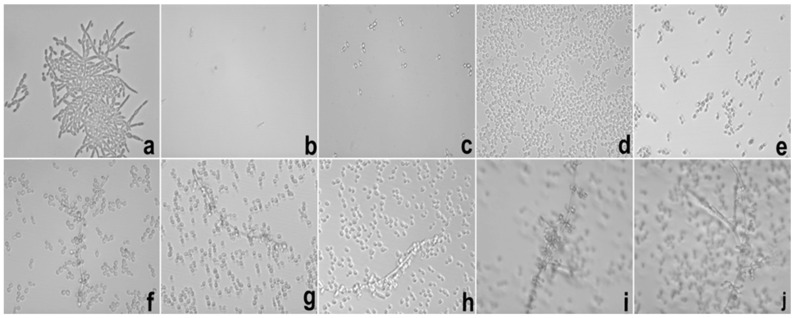
Photomicrography of 12 h of the hyphae inhibition test of *Candida albicans* (*C. albicans*) realized with E. Legend: (**a**) growth control; (**b**) amphotericin B 16 μg/mL; (**c**–**j**) E from 1000 to 7.8 μg/mL in descending order. E = Extract solution (without incorporation).

**Figure 6:**
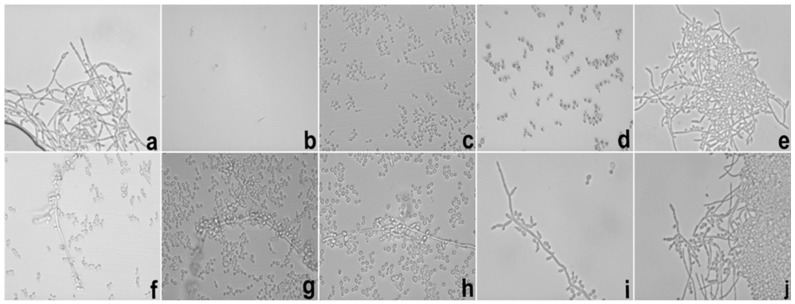
Photomicrography of 24 h of the hyphae inhibition test of *C. albicans* realized with E. Legend: (**a**) growth control; (**b**) amphotericin B 16 μg/mL; (**c**–**j**) E from 1000 to 7.8 μg/mL in descending order. E = Extract solution (without incorporation).

Figure 5 and Figure 6 show the test images from photomicrographs obtained by inverted light microscopy under 40× magnification with the results of the evaluation of the inhibitor effect of E targeting hyphae growth against *C. albicans* (ATCC 10231).

From the presented results, we could conclude that E was able to exercise hyphae inhibition in both periods. At 12 h, inhibition was observed at concentrations of 1000–250 μg/mL; however, this profile changed after 24 h of incubation, when the concentrations capable of inhibiting hyphae growth were 1000–500 μg/mL.

**Figure 7:**
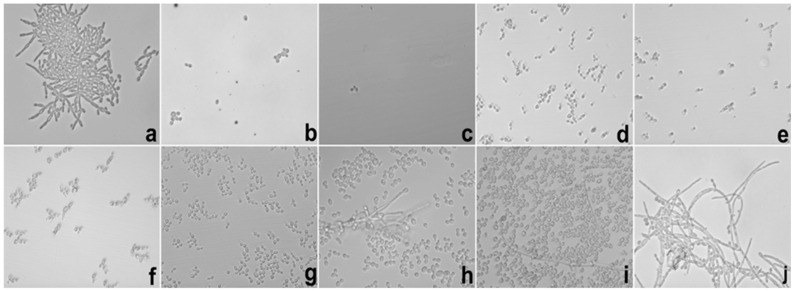
Photomicrography of 12 h of the hyphae inhibition test of *C. albicans* realized with SE. Legend: (**a**) growth control; (**b**) amphotericin B 16 μg/mL; (**c**–**j**) SE from 1000 to 7.8 μg/mL in descending order. SE = Formulation with extract.

**Figure 8:**
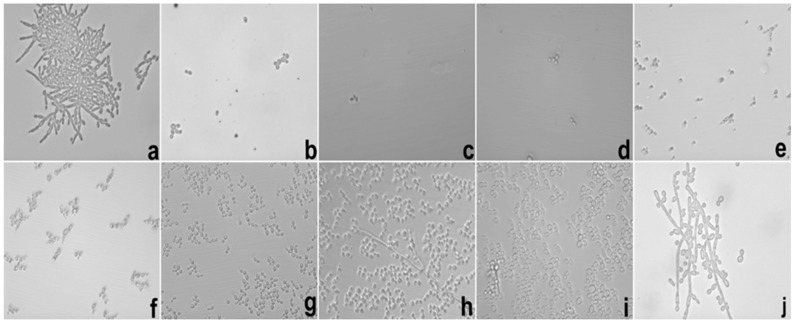
Photomicrography of 24 h of the hyphae inhibition test of *C. albicans* realized with SE. Legend: (**a**) growth control; (**b**) amphotericin B 16 μg/mL; (**c**–**j**) SE from 1000 to 7.8 μg/mL in descending order. SE = Formulation with extract.

The SE inhibition of hyphae occurred at a concentration 31.2 after 12 and 24 h of testing. These results demonstrated that the action of SE was superior to E. Figure 7 and Figure 8 show test images from photomicrographs obtained by inverted light microscopy under 40× magnification.

#### 2.2.3. In Vitro Assay of Inhibition of the Biofilm 

Table 4 shows the results of the in vitro test of biofilm formation inhibition against all of the *C. albicans* strains used in this study.

The results showed that E was not able to inhibit biofilm formation by all of the yeast strains, but SE exercised significant potential inhibition.

#### 2.2.4. Time Kill Assay

**Figure 9:**
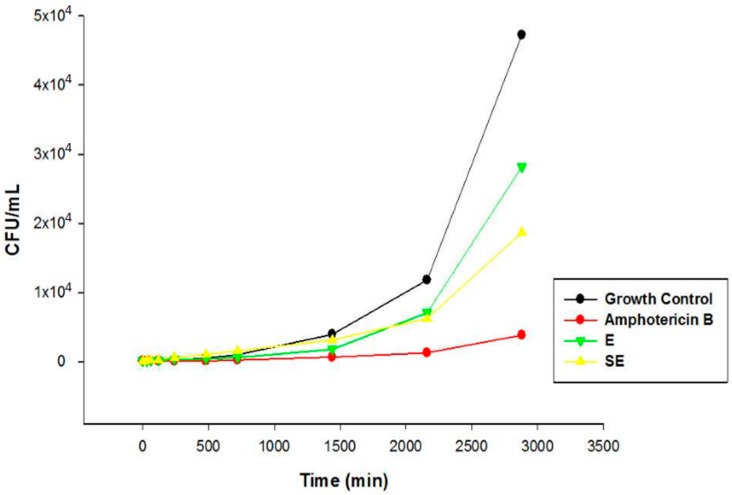
Time kill assay of ATCC 10231 strain. Legend: E = Extract solution (without incorporation); SE = Formulation with extract.

**Figure 10:**
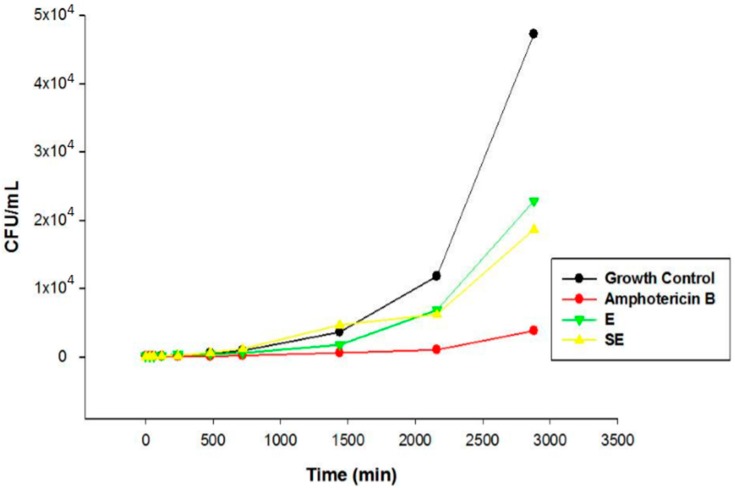
Time kill assay of CAV3 strain. Legend: E = Extract solution (without incorporation); SE = Formulation with extract.

Figure 9 and Figure 10 show the interference of the extract (both unloaded and loaded) on the growth of the strains tested over a 48-h time course.

Note that both strains were similar with regards to fungal growth time, which was maintained until the balanced time of 8 h; after this period, the CFU proliferation remained more constant in greater quantities.

#### 2.2.5. In Vivo Assay of Vulvovaginal Candidiasis Treatment

Table 5 and Table 6 show the number of infected animals and the fungal burden of all experimental groups in this study.

## 3. Discussion

The results obtained from the characterization of the system showed the applicability of the formulation S to be a precursor system of liquid crystal.

The formulations containing 5% and 10% AVM showed both a liquid and dark field, which was characteristic of microemulsion. The 30% and 50% AVM preparations showed Malta crosses (30%) and striae (50%), which were characteristic of the lamellar and hexagonal phases, respectively. The formulation with 100% AVM was more viscous and showed a hexagonal structure (striae) [19]. Then, a phase transition was observed for the region of the translucent viscous system when AVM was added to S (100%), indicating that the conformation was changed.

Therefore, based on these results, we proposed that the formulation could behave as a precursor system of liquid crystals because formation of the liquid crystalline system occurred following the addition of increasing amounts of mucus.

The rheological behavior of the formulations is desirable because, at the moment of the administration, the SE formulation will flow easily from the syringe, thereby facilitating its administration. Contact with vaginal mucus will increase the formulation viscosity, which allows it to stay in contact with the vaginal mucosa so it can release the extract for a longer time, thereby improving the clinical performance of the treatment.

Liquid crystalline hexagonal phases have been widely reported to be mucoadhesive systems due to their high viscosity; however, this property makes their applicability for vaginal administration difficult. Therefore, the system administration of a precursor Newtonian liquid crystal that will form a liquid crystalline mesophase that is mucoadhesive in situ represents one way to overcome this difficulty.

The S100 and SE100 formulations were able to interact more strongly with the vaginal mucosa and consequently remained for a longer time in the vaginal environment. 

Thus, the system designed here represents a novel system for vaginal release because it combines the advantage of forming a strong matrix liquid crystal in situ with high vaginal mucoadhesive strength [20].

The results of the in vitro determination of antifungal potential showed the antifungal effect exerted by the plant extract, which was shown to be active against all of the strains used in this study. According to the data presented in Table 3, we concluded that the incorporation of the extract in S showed promise because there were decreases in the MICs of the extract for all tested strains. These results may be related to the interaction characteristics with the fungal cell membranes exerted by S (i.e., the use of oleic acid as the oil phase of the formulation). Because oleic acid is a constituent of the cell plasma membrane, it can facilitate the passage of the active compound through the fungal membranes [21]. The polymer dispersion used as the aqueous phase of the formulation may also be related to the increased biological activity of E by promoting the controlled release of the active principle (i.e., the extract is deposited on the polymer chain and is thus released in a more gradual and controlled manner) [22].

The literature does not provide a consensus in terms of the MIC values obtained for natural products. Aligiannis and co-workers [23] considered MICs with values lower than 500 μg/mL to be potent inhibitors, MICs between 600 and 1500 μg/mL to be moderate inhibitors and MICs above 1600 μg/mL to be weak inhibitors. Webster and co-workers [24] established a satisfactory MIC as an MIC with a value equal to or less than 1000 μg/mL. In this sense, the results reported in this investigation are extremely important.

Secondary metabolites are the major molecules able to develop action against several pathogenic microorganisms by different action mechanisms. The cytoplasmic membrane is classified as the most common site of action of secondary metabolites, with action on this structure triggering with extravasation of cellular contents and, consequently, death of fungal. The interaction with genetic material and protein synthesis is also a predisposing factor in the promotion of therapeutic action. In this case, the contact of the genetic material with secondary metabolites promotes changes in DNA, results in ineffective transcripts promoting disorganization of vital functions of the cell [9].

The chemical characterization developed by Pacífico [12] and co-workers showed the presence of secondary metabolites in *S. nitens* extract such as flavones and xanthones. In this sense, the promising antifungal activity presented by extract in this study can be attributed by presence of these compounds. The MIC results were confirmed by Time kill assay, and showed that E and SE are able to control the microbial growth of the strains. Comparing the results between E and S in the assay revealed that the growth inhibitory action exerted by SE was higher than E, where the growth was controlled. After this period, the growth increased at a higher intensity but remained lower compared to the growth control at the end of 48 h, indicating a possible fungistatic mechanism for E and SE.

Besides of these important results, the potential hyphae inhibition exerted by E against *C. albicans* was superior when it was loaded into S, which could be explained using the same parameters reported in the MIC results. These data are important because the mechanism of cell infestation exercised by *C. albicans* in the host mucosa occurs mainly through the hyphae of the fungi in epithelial cells [25].

In the biofilm inhibition assay, it was observed that the adhesiveness of S increased its contact with the *C. albicans* biofilm, causing the direct and more intense release of SE with the microbial surface; this finding may explain the inhibition observed in relation to E. It is possible that the observed action is linked to the increased permeability of the fungal membrane that promoted an increase in the substantivity of E. Likewise, it is also possible that the mucoadhesive property interfered directly with the inhibition of the biofilm because it maintained direct contact with the system containing E in a uniform manner with more intense delivery and was fixed in place. The mucoadhesive components (PD and PRO) may have been the main action behind this result because we were able to fix the formulation containing the extract directly onto the biofilms formed in the well of the microplate, which in turn were presented as fixed and uniform biofilms. The search for new compounds that promote the inhibition of fungal biofilms is a focus of research in several countries to stimulate the improvement of drug administration [2]. The use of mucoadhesive drug delivery systems on biofilms is more suitable when employing polymers that promote direct adhesion with the biofilm as the most promising and effective route for this treatment [26].

The development of a drug delivery system that is reliable, effective, and safe for treatments against diseases is a goal for various researchers. Furthermore, drug delivery systems should enable the distribution of the drug through the intended route of administration and that prioritize the best drug–receptor interaction and the reduction of harmful effects [14]. So, an ideal drug delivery system should provide a targeted therapy that will allow effective concentrations of a drug to reach the disease site without exposing other tissues to toxicity. In this way, therapeutic treatment employing liquid crystals as drug release from vaginal diseases caused by pathogenic or opportunistic microorganisms is classified as potentially promising, especially because they can incorporate water from the vaginal mucus to promote structural changes in the system, resulting in a liquid crystalline phase and thereby optimizing the efficiency of the drug [27].

The use of *S. nitens* in the treatment of VVC caused by *C. albicans* was studied by Araújo and co-workers [13]. The authors reported the therapeutic action of the plant extract when it was incorporated in a conventional formulation. The present study showed that the incorporation of the extract in a nanotechnology-based drug delivery system showed superior results compared to the previous study, thereby indicating that S substantially increased the action of the bioactive agent.

In recent years, the use of nanotechnology for drug delivery systems for the incorporation of plant extracts has been shown to be important for vaginal applications [14]. Bonifácio and co-workers [28] showed that the antifungal potential of the ethanolic extract of the leaves of *Astronium urundeuva* was improved with the incorporation into a nanostructured lipid system (lipid microemulsion) in the treatment of VVC. The results showed by these authors proved that the therapeutic profile in vivo model using the system cured the animals with only six days of treatment. However, the unloaded extract was not effective during the eight days of treatment.

In this study, SE exerted a cured profile on the animals of experimental groups (7 and 13) after only 2 days of treatment, which may be due to the interaction with the fungal membranes described in this work. Therefore, the inhibitory profile in relation to the mucoadhesive properties was presented by S. Infected animals produced excess vaginal discharge that came into contact with the system and promoted membership in the vaginal mucosa, there by stimulating a direct interaction of the active principle with the mucous membranes and triggering a direct release at the desired site of action [29,30,31].

The activity observed with E was significant because it promoted healing of the animals infected with the ATCC strain four days after administration. The animals infected with CAV3 were healed after six days of treatment. The late action compared to the incorporated extract can be explained based on the low viscosity of the vehicle (DMSO 20%) used to solubilize E because, at the time of administration, the animals cast out the contents deposited in the vaginal canal after the application, which led to a low concentration of E in the intravaginal environment.

The therapeutic action with the standard drug (amphotericin B) was inferior to the action shown by the non-incorporated and incorporated extracts, thereby demonstrating that the use of E in both forms is more promising. Reports of the importance of this result concerning the use of amphotericin B are limited due to its nephrotoxicity-related reactions [32].

We highlighted the results of the therapeutic profile shown by the extract loaded into S because it may be related to the adhesion property.

The objective of the present system was based on the fixation characteristics of the vaginal epithelium; thus, materials with adhesive properties were used. The aqueous phase of the system (polymeric dispersion) was also developed to promote adhesion in the vaginal environment. The adhesive behavior is due to physical and chemical processes (i.e., hydrophobic interactions, hydrogen bonds and van der Waals forces) [17]. In addition to this characteristic, the use of polymers in the aqueous phase was intended to promote slower liberation and a controlled manner of the plant extract.

The adhesive materials in the pharmaceutical formulations may be hydrophilic molecules of natural or synthetic origin that contain numerous organic components (i.e., carboxyl groups, hydroxyl groups and amines) that form chemical bonds with the biological surface and promote the increase of permeability (i.e., membrane cells) [33].

The choice of surfactant (PEG-5 Ceteth-20 Procetyl^®^, Wickliffe, OH, USA) and the constituent of aqueous phase was based on adhesive characteristics because they can come into contact with substances that has water to promote the adhesiveness. Therefore, we aimed to trigger adhesion when the system came into contact with vaginal mucus.

Systems that have polymeric network compositions, the drug may be homogenously dispersed in the polymer matrix or adsorbed on their surface or within a reservoir. This phenomenon involves the liberation of the same physical and chemical processes, such as water penetration into the matrix, diffusion of the drug through the pores of the matrix, polymer degradation or a combination of the last two mechanisms [14]. 

In another study, the use of polymeric dispersion (chitosan) as aqueous phase in liquid crystalline system for incorporation of curcumin showed antifungal activity against *C. albicans* according to in vitro tests. The authors proved that adhesive property exercised by this polymer was important to promote the intense contact of the system with the fungal membrane cells. This contact may be responsible for the increase of membrane permeability that facilitated the entrance of active constituents within the intracellular environment [27].

## 4. Material and Methods

### 4.1. Materials

Polyoxypropylene (5) polyoxyethylene (20) cetyl alcohol (PPG-5-CETETH-20) was purchased from Croda (Campinas, Sao Paulo, Brazil). Oleic acid was purchased from Synth (Diadema, Sao Paulo, Brazil). Polycarbophil^®^ and Carbopol^®^ 974P was purchased from Lubrizol^®^ (Wickliffe, OH, USA) Advanceds materials (Cleveland, OH, USA). Sterile fetal bovine serum was purchased from Laborclin^®^ (Pinhais, Paraná, Brazi) laboratory products, Pinhais, Paraná-Brazil. The high-purity water was prepared with a Millipore Milli-Q Plus purification system, and its resistivity was 18.2 MΩ-cm. Sabouraud Dextrose Ágar, Both and supplemented with chloramphenicol were purchased from Difco^®^-Becton (Franklin Lakes, NJ, USA) Dickinson and Company Sparks (Le Pont de Claix, France). Methanol was purchased from Merck^®^ (Darmstadt, Germany). Triethanolamine, Mucin from porcine stomach type II, amphotericin B, fluconazole, estradiol, cyclophosphamide, XTT sodium salt, triphenyltetrazolium chloride (TTC), gentamicin were purchased from Sigma Aldrich^®^ (Steinheim, North Rhine-Westphalia, Germany). 

### 4.2. Vegetable Plant and Preparation of the Extract

The collection of plant material was performed in Serra do Jalapão in the state of Tocantins, Brazil, after owner of the land gave permission to conductivity the study. A number of SPF 189975 voucher specimen was deposited in the IB-USP Sao Paulo, Brazil.

The plant extract was prepared by an exhaustive extraction method consisting of simple percolation using methanol as the solvent [34]. The extract was concentrated under reduced pressure by rotary evaporation (48 h) at a temperature below 40 °C and then lyophilized.

### 4.3. Development of the Liquid Crystal Precursor Mucoadhesive System (LCPS)

To prepare the LCPS, we used oleic acid (OA) as the oil phase and polyoxypropylene (5) polyoxyethylene (20) cetyl alcohol (PPG-5-CETETH-20)—Procetyl (PRO) as the surfactant. The aqueous phase was comprised of a 5% polymer dispersion (PD) synthesized from two polymers: (0.5% Polycarbophil^®^ (PP) and 0.5% Carbopol C974P^®^ (CP)) suspended in Milli-Q^®^ water with mechanical stirring and the pH adjusted to 7.0 with triethanolamine (TRI).

For preparation of the ternary phase diagram [19], different proportions (0%–100% *w*/*w*) of each phase of the system were mixed at room temperature (25 ± 0.5 °C) with stirring, resulting in the construction of the ternary phase diagram with 36 formulations. All formulations that were not at pH 7.0 were adjusted with TRI. All systems were visually classified as Transparent Liquid System (TLS), Transparent Viscous System (TLS), Translucent Liquid System (TrLS), Translucent Viscous System (TrVS), Viscous and Opaque System and Phase Separation (PS). Thus, it was possible to delineate the different regions of the phase diagrams. From this data, the regions of the systems used for physical-chemical characterization were selected. The choice of the optimal formulation for the development of antifungal in vivo and in vitro screening was based on the characteristics observed in each of the 36 formulations. One formulation was obtained to produce a stable liquid with the lowest concentration of surfactant and thus reduce the potential toxicity of the formulation and demonstrate that the incorporation of the water-formulation caused increased viscosity, thereby simulating the vaginal environment. 

Formulation S (composed of 40% oil phase, 40% surfactant and 20% aqueous phase) was selected as the precursor system of the mucoadhesive liquid crystal. The E was incorporated into S, which resulted in the formulation SE.

### 4.4. Structural Characterization of the System and Pharmacotechnique Analysis

#### 4.4.1. Polarized Light Microscopy (PLM)

A drop of each formulation was placed onto a glass slide covered with a cover slip and then examined through the polarized light microscope (Olympus BX41) coupled with the QColor3 Chamber (Olympus America Inc., New York, NY, USA) at 25 ± 0.5 °C. The isotropic or anisotropic behavior of the samples was noted. Photomicrographs were taken at a magnification of 20,000×.

#### 4.4.2. Assay with Artificial Vaginal Mucus (AVM)

To verify the property of the precursor of the liquid crystal, we developed artificial vaginal mucus (AVM) prepared as described by Owen and Katz [35]. For the preparation of 1 L of AVM, each constituent was weight (g), is as follows: NaCl, 3.51; KOH, 1.40; Ca(OH)_2_, 0.222; bovine serum albumin, 0.018; lactic acid, 2.00; acetic acid, 1.00; glycerol, 0.16; urea, 0.4; and glucose, 5.0. After complete solubilization, 15 g mucin was added. The pH was adjusted to 4.2 using 0.1% HCl. The evaluation of the AVM effect in S and SE (2 mg/mL) were assessed when 5%, 10%, 30%, 50% and 100% of the AVM was added in relation to the initial mass (2 g). Representative samples were analyzed by PLM, and structural changes were elucidated. This test allowed us to evaluate the behavior exercised by the in vitro system developed to simulate the formation of liquid crystals in contact with AVM.

#### 4.4.3. Rheological Analysis

After interference analysis, the amount of AVM that promoted better organization of selected formulations was chosen; therefore, 100% AVM was added to the formulations. Rheological testing was also performed with S and SE, resulting in S + 100% AVM (S100) and SE + 100% AVM (SE100).

Continuous flow was analyzed on a controlled-stress AR2000 (TA Instruments, New Castle, DE, USA) equipped with parallel plate geometry (40 mm diameter) and a sample gap of 200 μm at 37 ± 0.1 °C in triplicate. Samples of the systems were carefully applied to the lower plate, thereby ensuring that sample shearing was minimized, and allowed to equilibrate for 3 min prior to analysis.

Continual testing was performed using a controlled shear rate procedure in the range from 0.01 to 100 s^−1^ and back, each stage lasting 120 s with an interval of 10 s between the curves. The consistency index and flow index were determined from the Power law described in Equation (1) for the quantitative analysis of flow behavior [19].

(1) $$\tau =k\cdot \gamma^{\eta }$$

where “τ” is the shear stress, “*γ*” is the shear rate, “*k*” is the consistency index and “*η*” is the flow index.

#### 4.4.4. In Vitro Evaluation of Mucoadhesive Force

We used the vaginal mucosa of pigs courtesy of a local producer and the use was approved by the Animal research committee CEUA-UNESP. Freshly excised pig vaginal mucosa was frozen at −30 °C. A section with a 2 mm thickness was excised from the inner part of the surface of the frozen vaginal mucosa and fitted on the mucoadhesion test rig. Then, 50 μL of AVM was applied to the surface of the tissue prior to the experiment [19]. The samples were packed into shallow cylindrical vessels. The test began by lowering the analytical probe that contained the skin at a constant speed (1 mm·s^−1^) onto the surface of the sample. The mucosa and the sample were kept in contact for 60 s; no force was applied during this interval. After 60 s, the mucosa was drawn upwards (0.5 mm·s^−1^) until the contact between the surfaces was broken. The mucoadhesive force of the samples was measured as the maximum detachment force of the resistance to the withdrawal of the probe, which reflected the mucoadhesion characteristic. Seven replicates were analyzed at 37 ± 0.5 °C.

### 4.5. In Vitro Antifungal Assays

#### 4.5.1. Fungal Strains

One strain of *C. albicans* (ATCC 10231) and five clinical strains (CAV1, 2, 3, 4 and 5) were used in the in vitro assays. The clinical strains were donated to the Microbiology Laboratory of the Faculty of Medicine of Sao Jose do Rio Preto for purposes of scientific research through a written consent of the donors and with was approved by the Human Research Ethics Committee CEP-FAMERP (Protocol number 152/2006).

Table 7 shows the strains used in this study with their respective general considerations.

#### 4.5.2. Determination of the Minimum Inhibitory Concentration (MIC) 

In the MIC, determination was performed by the dilution in microplate technique [36] with modifications. The solutions of E and SE (100 μL) were added at concentrations ranging from 1000 to 7.8 μg/mL (serial dilution). Yeast cultures incubated for 48 h were adjusted to 10^3^ CFU/mL which was then distributed into each well of the microplate. Amphotericin B and fluconazole were used as the positive controls. Additional controls also included the culture medium, yeast growth, E, solvent (DMSO) and formulation S. The microplates were incubated at 37 °C for 48 h. 

The 20 μL of an aqueous 2% solution of 2,3,5-triphenyltetrazolium chloride (TTC) was used as developer [37] and the microplates were incubated at 37 °C for 2 h. The assays were carried out in triplicate.

#### 4.5.3. Analysis of the Inhibitory Profile of Hyphae in *C. albicans*

The *C. albicans* (ATCC 10231) culture was grown for 24 h to obtain filamentous yeasts. The yeasts at a concentration of 2.5 × 10³ cells/mL in PBS (pH 7.2). A total of 20 μL of this suspension was added to microplate wells containing RPMI 1640 medium with 10% fetal bovine serum and gentamicin (1%). E and SE were evaluated at concentrations ranging from 1000 to 7.81 μg/mL. After 12 and 24 h, a reduction in hyphae growth was observed under the inverted light microscope (400×). Amphotericin B (16 μg/mL) was used as the positive control; additional controls included fungal growth, DMSO, sterile S and culture medium [13].

#### 4.5.4. In Vitro Assay of Biofilm Inhibition

The biofilm adhesion method was performed as described by Pitangui et al. [38] with modifications. The samples were incubated under rotation at 37 °C for two hours at 80 rpm. After the pre-adhesion period, the supernatant was removed and 100 μL of RPMI medium was added to each microplate well; then, incubation proceeded at 37 °C for 48 h, with the RPMI renewed after 24 h. After the incubation period, the supernatant was removed and the wells were washed with 100 mL of 0.9% saline solution. A total of 100 mL of E and SE were added at concentrations of 20, 10, 5, 2.5, 1.2 and 0.6 mg/mL, and the microplates were re-incubated for 48 h at 37 °C. The 2,3-bis (2-methoxy-4-nitro-5-sulfophenyl)-5-[carbonyl(phenylamino)]-2H-tetrazolium hydroxide (XTT^®^) was used as the developer are shown the reduction of the medium. 

#### 4.5.5. Time Kill Assay

This test was performed with the standard strain (ATCC 10231) and the clinical strain that was most sensitive in the MIC assay (CAV 3). In a test tube containing culture medium and yeast (2.5 × 10^3^ cells/mL), we added 1 mL of E and SE at a concentration that was 2× the obtained MIC and the mixture was incubated at 37 °C. The aliquot of the contents were diluted in sterile PBS (1:1) at ratio 0, 0.5, 1, 2, 4, 8, 12, 24, 36 and 48 h. This suspension was seeded onto the surface of SDA and the colonies were counted after 48 h of incubation [39].

### 4.6. In Vivo Antifungal Assay

#### 4.6.1. Experimental Assay of Vulvovaginal Candidiasis Treatment

This assay was approved by the Animal research committee CEUA-UNESP (Protocol number 34/2013).

The assays were performed with two strains of *C. albicans*: a clinical isolate (CAV3- most sensitive in the MIC determination) and the standard strain (ATCC 10231). *Wistar* female rats were employed (*Rattus novergicus*) (8 weeks old, 200–300 g). The animals were maintained throughout the experiment in the bioterium of the School of Pharmaceutical Sciences of Araraquara—UNESP, with adequate temperature and ventilation under a 12-h light/dark cycle and free access to water and food during the course of all experiments. The animals were housed in cages with previously sterilized wood shavings and were acclimated to the experimental room for 7 days prior to the start of the procedures.

We used the in vivo experimental model of Araújo et al. [13] with modifications. The animals were subjected to a state of immunosuppression by the administration of cyclophosphamide (0.3 mL−50 mg/kg) in a single intraperitoneal dose. To obtain the pseudo-estrus state, the rats were injected with a 0.1 mL solution of estradiol (0.2 mg/mL) subcutaneously on the day of immunosuppression and 10 days later. The hormonal status was verified by microscopic analysis of cell morphology found in the vaginal fluid of animals obtained by washing with 0.1 mL of PBS solution; the presence of cornfield anucleate epithelial cells indicated the pseudo-estrus phase [40]. After adjusting the estrous cycle of all animals, they were infected intravaginally with a suspension of 5.0 × 10^8^ cells/mL of *C. albicans* (ATCC 10231 and CAV 3) prepared in PBS by injecting 0.1 mL of the fungal suspension with the aid of a micropipettor with a sterile tip. Two days after inoculation, vaginal washings were performed with 0.1 mL of sterile PBS buffer solution. The washes were subjected to microscopic examination and cultured on sabouraud dextrose agar with chloramphenicol (SDA + clo) plates which were incubated at 37 °C for 48 h. The presence of cells or free budding yeast observed under a light microscope and the growth of CFUs in the culture medium were considered positive for infection.

#### 4.6.2. Experimental Groups and Therapeutic Treatment

We used 13 experimental groups consisting of 6 animals each (*n* = 78). Table 8 shows the experimental groups and their respective treatment parameters. 

The concentration of vegetal extract used in the therapeutic groups was based in the MIC value obtained in the in vitro determination of antifungal potential by microdilution technique. A value of 2× MIC was employed as a therapeutical dose [17,38].

The treatments used in the assays were performed twice daily for 8 days.

#### 4.6.3. Analysis of the Effectiveness of Treatment

Microscopic analysis and culture of vaginal fluids were performed to determine the vaginal fungal burden. On days 2, 4, 6 and 8 of the treatment period, the animals were subjected to the collection of vaginal fluids which were obtained by intravaginal washing with 0.1 mL of PBS solution (pH 7.4) with the aid of a sterile micropipettor with sterile tips. Microscopic analyses were performed to verify the presence or absence of yeast in the vaginal environment. The washes were also cultured in SDA + clo plates which were incubated at 37 °C for 48 h and quantified by the number of CFU on each day to analyze the treatment.

#### 4.6.4. Analysis of Recurrence of Infection and Euthanasia

Eight days after the treatment period, the animals were subjected to recurrence of the infection to identify and diagnose infectious states that may eventually emerge after the treatment period. To this end, vaginal fluids were collected daily over a 7 day period for microscopic analysis and culture as described above for the analysis of the efficacy of treatment. The animals were euthanized by intoxication in a CO_2_ chamber. 

Statistical data were analyzed using ANOVA. We used the Tukey test to compare the results of the treatments and the Dunnett test to compare the results of the treatment and control.

## 5. Conclusions

This study confirms the antifungal potential of *S. nitens* extract and shows that the incorporation into the drug delivery system is important for the increase of its pharmacological parameters. The use of the LCPS (S) proves to be suitable for the treatment of VVC which make them more efficient than E unloaded. The improvement in antifungal activity can be based on the mucoadhesive properties of the formulation and the components that might have facilitated the interaction with the fungal cells. Besides the prophylactic potential showed in our previous studies, in this study we conclude that the incorporation of *S. nitens* in LCPS is able to promote the treatment of VVC caused by *C. albicans*.

## Figures and Tables

**Table 1:** Results of the evaluation of precursor behavior the formulation of liquid crystals by Polarized Light Microscopy (PLM).

Test	Viscosity	Structure Viewed	Mesophase
Formulation + 5% of AVM	+	Dark field	Microemulsion
Formulation + 10% of AVM	++	Dark field	Microemulsion
Formulation + 30% of AVM	+++	Cross of Malta	Lamellar
Formulation + 50% of AVM	++++	Striae	Hexagonal
Formulation + 100% of AVM	+++++	Striae	Hexagonal

+ = viscosity; AVM, Artificial Vaginal Mucus.

**Table 2:** Flow behavior (*n*) and consistency index (K) of the formulations.

Formulations	*n*	K
SE100	0.30	17.64
S100	0.23	55.11
S	1.0	0.059
SE	1.0	0.069

Each value represents the mean (±SD) of three replicates. S = Formulation; SE = Formulation with extract; S100 = Formulation + 100% of AVM; SE100 = Formulation with extract + 100% of AVM.

**Table 3:** Minimum Inhibitory Concentration (MIC) of E and SE against *Candida albicans* (*C. albicans*).

Sample Analyzed	MIC ^a^			
	E	SE	Fluconazole	Amphotericin B
ATCC 10231	250	62.5	R	0.12
CAV 1	125	62.5	R	0.12
CAV 2	250	62.5	R	0.12
CAV 3	125	31.2	R	0.12
CAV4	125	62.5	R	0.50
CAV5	250	62.5	R	0.25

^a^ Values in μg/mL; (R) resistance. E = Extract solution (not loaded); SE = Formulation with extract.

**Table 4:** Results of inhibition biofilm assay.

*C. albicans* Biofilm	Inhibition ^a^				
	SE	E	AMB	DMSO	S
ATCC 10231	1.25	>20.0	4.0	-	-
CAV 1	10.0	>20.0	8.0	-	-
CAV 2	20.0	>20.0	4.0	-	-
CAV 3	10.0	>20.0	8.0	-	-
CAV 4	1.25	>20.0	16.0	-	-
CAV 5	1.25	>20.0	16.0	-	-

^a^ values in mg/mL; (-) without inhibition. AMB = amphotericin B; E = Extract solution (not loaded); SE = Formulation with extract; DMSO = dimethyl sulfoxide (20%); S = Formulation.

**Table 5:** Fungal loads (CFUs) obtained from the culture of vaginal fluid collected during the treatment period for therapeutic treatment against *C. albicans* ATCC 10231.

Groups	Treatment			
	Day 2	Day 4	Day 6	Day 8
Positive control (infection)	8030.0 ± 254.6	5830.0 ± 56.6	6700.0 ± 141.4	8123.0 ± 113.1
Positive control (tetracycline + amphotericin B)	2507.0 ± 42.4	947.0 ± 84.9	160.0 ± 0.0	0.0 ± 0.0
Solvent control (DMSO)	6007.0 ± 42.4	6447.0 ± 70.7	5303.0 ± 28.3	8043.0 ± 28.3
Treatment 1 (E)	170.0 ± 0.0 ^a,^*	253.0 ± 0.0 ^a,^*	0.0 ± 0.0 ^a^	0.0 ± 0.0 ^a^
S formulation	4587.0 ± 28.3	5793.0 ± 28.3	6587.0 ± 84.9	6287.0 ± 53.5
Treatment 2 (SE)	0.0 ± 0.0 ^a,^*	0.0 ± 0.0 ^a,^*	0.0 ± 0.0 ^a^	0.0 ± 0.0 ^a^

^a^ The same letter denotes non-significant differences among groups according to parametric post hoc test (*p* < 0.05—Tukey test); * significant difference (*p* < 0.05) when compared with positive control group (tetracycline + amphotericin B—Dunnett test).

**Table 6:** Fungal loads (CFUs) obtained from the culture of vaginal fluid collected during the treatment period for therapeutic treatment against CAV3.

Groups	Treatment			
	Day 2	Day 4	Day 6	Day 8
Positive control (infection)	7203.3 ± 42.4	16716.0 ± 172.1	16683.3 ± 14.1	16130.0 ± 28.3
Positive control (tetracycline + amphotericin B)	7270.0 ± 14.1	4256.7 ± 141.4	2143.3 ± 200.3	416.7 ± 118.5
Solvent control (DMSO)	5726.7 ± 56.6	8356.7 ± 127.3	8936.7 ± 198.0	8563.3 ± 0.0
Treatment 1 (E)	546.7 ± 30.0 ^a^	110.0 ± 91.9 ^a^	63.3 ± 44.8 ^a^	0.0 ± 0.0 ^a^
S formulation	5340.0 ± 28.3	10470.0 ± 28.3	10683.3 ± 186.2	9476.7 ± 80.9
Treatment 2 (SE)	0.0 ± 0.0 ^a^	0.0 ± 0.0 ^a^	0.0 ± 0.0 ^a^	0.0 ± 0.0 ^a^

^a^ The same letter denotes non-significant differences among groups according to parametric post hoc test (*p* < 0.05—Tukey test); * significant difference (*p* < 0.05) when compared with positive control group (tetracycline + amphotericin B—Dunnett test).

**Table 7:** *Candida albicans* strains.

*C. albicans* Strains	Origin ^a^	Resistance Profile ^b^	Symptomatology
ATCC 10231	Pulmonary	ketoconazole, fluconazole and itraconazole	Not described
CAV1	Vaginal	ketoconazole, fluconazole, itraconazole and nystatin	Symptomatic
CAV2	Vaginal	ketoconazole, fluconazole and itraconazole	Asymptomatic
CAV3	Vaginal	ketoconazole, fluconazole, itraconazole and nystatin	Symptomatic
CAV4	Vaginal	ketoconazole, fluconazole and itraconazole	Symptomatic
CAV5	Vaginal	ketoconazole, fluconazole and itraconazole	Asymptomatic

^a^ All strains are of human origin; ^b^ according to microdilution tests (CLSI protocol).

**Table 8:** Experimental groups of in vivo treatment assay of vulvovaginal candidiasis (VVC).

Experimental Group	Specification	Treatment *
Group 1	Negative control	PBS solution sterile
Group 2	Positive control-ATCC 10231	PBS solution sterile
Group 3	Positive control with antifungal-ATCC 10231	0.1 mL antifungal cream (amphotericin B and tetracycline)
Group 4	Control of solvent-ATCC 10231	0.1 mL solution with 20% of DMSO
Group 5	Treatment 1-ATCC 10231	0.1 mL of E (500 μg/mL)
Group 6	Control of S-ATCC 10231	0.1 mL of S
Group 7	Treatment 2-ATCC 10231	0.1 mL of SE (500 μg/mL)
Group 8	Control of infection-CAV3	PBS solution sterile
Group 9	Positive control with antifungal-CAV3	0.1 mL antifungal cream
Group 10	Control of the solvent-CAV3	0.1 mL solution with 20% of DMSO
Group 11	Treatment 3-CAV3	0.1 mL of E (500 μg/mL)
Group 12	Control of S-CAV3	0.1 mL of S
Group 13	Treatment 4-CAV3	0.1 mL of SE (500 μg/mL)

* Treatment performed twice a day. S = Formulation; SE = Formulation with extract.
